# Adult phenotype and further phenotypic variability in SRD5A3-CDG

**DOI:** 10.1186/1471-2350-15-10

**Published:** 2014-01-16

**Authors:** Bülent Kara, Özgecan Ayhan, Gülden Gökçay, Nurdan Başboğaoğlu, Aslıhan Tolun

**Affiliations:** 1 Department of Pediatrics, Division of Child Neurology, Kocaeli University Medical Faculty, Kocaeli, Turkey; 2 Department of Molecular Biology and Genetics, Boğaziçi University, Istanbul, Turkey; 3 Department of Pediatrics, Division of Nutrition and Metabolism, Istanbul University Istanbul Medical Faculty, Istanbul, Turkey; 4 Department of Ophthalmology, Düzce University Medical Faculty, Düzce, Turkey

**Keywords:** SRD5A3, SRD5A3-CDG, CDG, Glycosylation defect

## Abstract

**Background:**

*SRD5A3* is responsible for SRD5A3-CDG, a type of congenital disorder of glycosylation, and mutations have been reported in 15 children. All the mutations are recessive and truncating.

**Case presentation:**

We present 2 brothers at the age of 38 and 40 years with an initial diagnosis of cerebellar ataxia. We found the candidate disease loci via linkage analysis using data from single nucleotide polymorphism genome scans and homozygous truncating mutation *SRD5A3* p.W19X, which was previously reported in 3 unrelated children, by exome sequencing.

Clinical investigations included physical and ocular examinations and blood tests. Severe ocular involvement with retinal bone spicule pigmentation and optic atrophy are the most prominent disabling clinical features of the disease. The serum transferrin isoelectric focusing (TIEF) pattern is abnormal in the patient investigated.

**Conclusion:**

Our patients are older, with later onset and milder clinical phenotypes than all patients with SRD5A3-CDG reported so far. They also have atypical ocular findings and variable phenotypes. Our findings widen the spectrum of phenotypes resulting from *SRD5A3* mutations and the clinical variability of SRD5A3-CDG, and suggest screening for *SRD5A3* mutations in new patients with at least a few of the clinical symptoms of SRD5A3-CDG.

## Background

Congenital disorders of glycosylation (CDG) are multisystemic disorders caused by defects in protein glycosylation. Since the first clinical description in 1980, about 60 CDG types with a very broad spectrum of clinical presentations have been reported [1,2]. *SRD5A3*, encoding steroid 5-alpha-reductase 3, is responsible for a rare CDG, namely, SRD5A3-CDG [CDG1Q; MIM 612379] [3,4]. So far, 15 children with SRD5A3-CDG in 12 families carrying 10 different recessive mutations and with variable phenotypes have been reported [3-7]. The disorder is characterized by severe early visual impairment and variable ocular anomalies including coloboma, optic nerve hypoplasia, congenital cataract, and glaucoma, as well as some degree of intellectual disability. Other clinical features, such as cerebellar ataxia and cerebellar malformations, ichthyosiform skin disorders, hypertrichosis, congenital heart defects, and abnormal coagulation vary considerably not only among families but also within a family [5]. Although the transferrin isoelectric focusing (TIEF) pattern is generally abnormal in affected individuals, one patient with SRD5A3-CDG was reported to have a normal pattern [5]. Therefore, even in the absence of an abnormal TIEF pattern, the clinically distinctive features of SRD5A3-CDG warrant genetic testing. Kahrizi syndrome is a similar disease and has been described in a single family. It has been characterized by intellectual disability, coloboma, cataract, and kyphosis but a normal TIEF pattern [8]. It was later shown to result from a homozygous truncating *SRD5A3* mutation, indicating that SRD5A3-CDG and Kahrizi syndrome are allelic disorders [9]. The affected siblings were aged 40 years and older. In contrast, all patients with SRD5A3-CDG reported so far have been children.

## Case presentation

### Family

The parents were second cousins. They had 2 affected sons and 4 healthy daughters. Peripheral blood samples were obtained after written informed consent, and DNA was isolated from leukocytes by a standard protocol.

### Genetic analyses

Single nucleotide polymorphism (SNP) genome scans were performed for the parents and the 2 affected sibs using an Illumina 370 Duo chip. Linkage analysis for calculating multipoint logarithm of the odds (LOD) scores was performed with Allegro using SNPs at intervals of 0.07 cM and sets of 100, and assuming recessive inheritance and a disease allele frequency of 0.001. Four candidate loci yielded the highest score of 2.66 (Additional file 1). The largest locus was maximally 52 Mbp at chromosome 4q12-24. The others were 0.55 to 5.5 Mbp at 6q25.1-25.2 (maximally between rs912561 and rs1407490), 12p13.31-p13.1 (rs1868798 and rs873218), and 22q12.1 (rs1547358 and rs5752043). We considered the largest locus (4q12-24) the most likely disease locus. The region with shared homozygosity was delineated by rs2067951 and rs11733406 (55,805,555 and 107,751,089 bp) and harbored 437 genes [10]. At the time, there were no similar diseases mapped to any of the loci. The abundance of the genes at the loci discouraged us from applying the candidate gene approach, and we launched exome sequencing.

A DNA sample from the older brother was subjected to exome sequencing analysis, aiming to cover 95% of the CCDS database, as previously described [11]. Ten novel exonic variants were found at 4q12-24. The best candidate responsible for the disease was the homozygous mutation c.57G > A (p.W19X) in *SRD5A3* exon 1, converting codon 19 (tryptophan) to a termination codon. We validated the mutation by Sanger sequencing (Additional file 2). The mutation segregated with the disease in the family. Fifty-six unrelated individuals from the population were screened for the mutation by single strand conformational polymorphism analysis and found not to carry it. The mutation was reported in 3 unrelated children with SRD5A3-CDG [4,6].

### Clinical data

**Figure 1:**
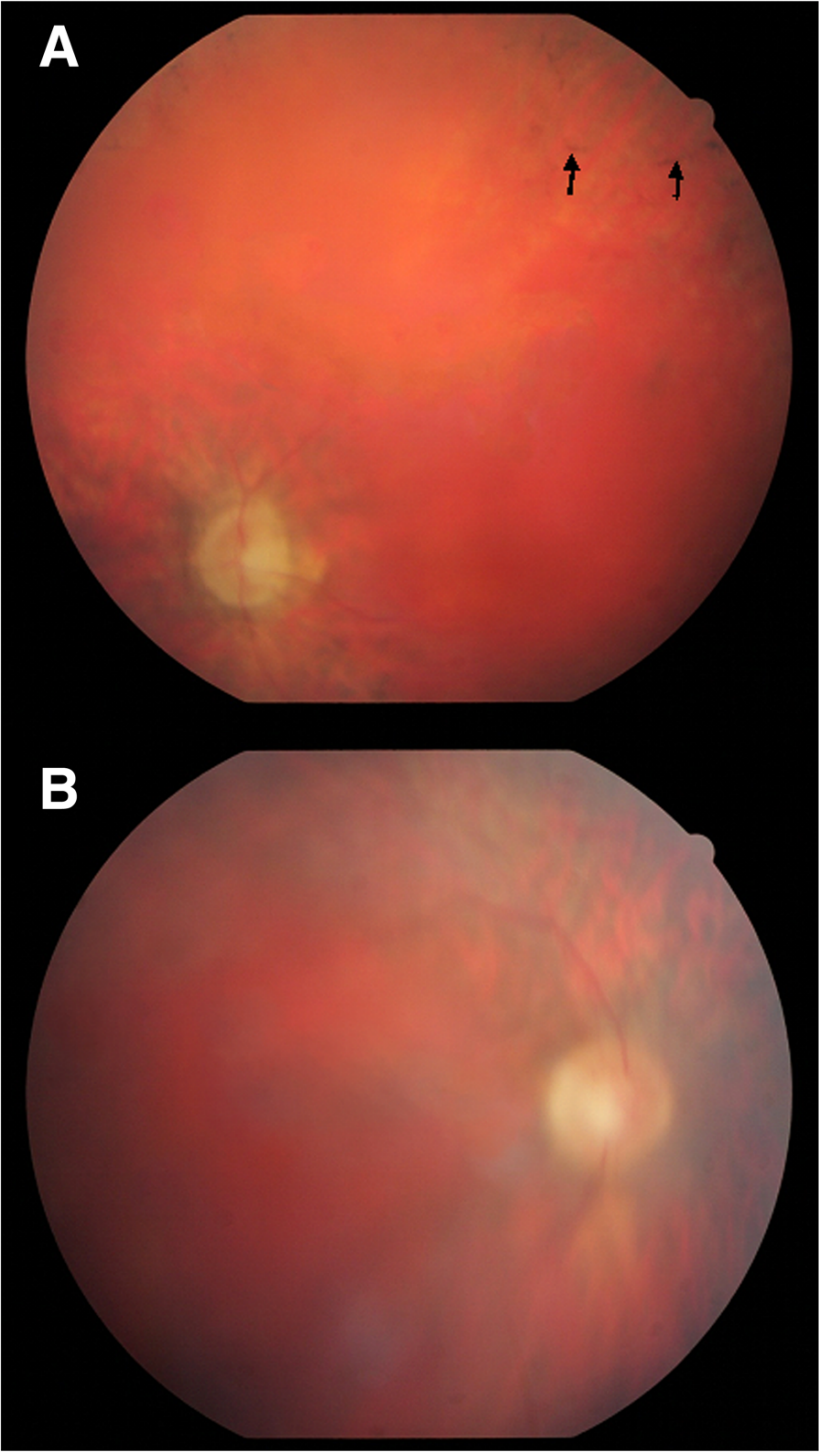
**Ocular findings. A**: Waxy pallor of the optic disc with attenuated retinal vessels and bone spicule-shaped pigment deposits in the periphery (indicated with arrows) in the older patient. **B**: Blurry fundus due to vitreous degeneration and a posterior subcapsular cataract in the younger brother.

The affected brothers were 38 and 40 years old and had complaints of abnormal gait and visual impairment, and the initial diagnosis five years ago was diagnosed with cerebellar ataxia. According to the parents, their prenatal, natal, and postnatal histories were uneventful. They walked and spoke single words by the age of 2 years. Whether they had childhood ichthyosiform dermatitis or coagulation abnormalities could not be assessed with certainty. Progressive ataxic gait and visual impairment began in early childhood. The older brother had a relatively more severe phenotype and could not complete primary school due to intellectual disability and ophthalmologic problems. At present, he lives with his parents, needs help with activities of daily living partly due to ophthalmologic problems, and does not have a job. The younger brother was able to complete primary school but not high school mainly due to ophthalmologic problems. At present, he works in an office and can use a computer. He is married and has 4 healthy children. Recent examinations of both patients revealed ataxic gait, dysmetria, dysdiadochokinesia, and scanning speech. Muscle tonus was normal, deep tendon reflexes were increased, and Achilles clonus was present. No pathologic reflex was observed. Cranial nerves were intact. The mental performance of the younger patient seemed much better. His sensory examination was normal and visual acuity was at the level of counting fingers from 25 cm. The older brother’s visual acuity was at the level of awareness of hand movements right in front of his face. In both patients, eye examination showed bilateral horizontal nystagmus, bilateral posterior subcapsular cataract, bilateral optic disk hypoplasia, thinning of the retinal arterioles, bilateral optic atrophy, and retinal bone spicule pigmentation (Figure 1). The younger patient also had 30–40° exotropia, bilateral keratoconus, and degenerated vitreous. Neither of the patients had glaucoma, dysmorphism, or organomegaly. Skin examinations were normal. The upper extremities of the older brother were slightly short. Whole blood count, coagulation studies, urine analysis, and serum biochemistry were performed for him, and the results were unremarkable.

### Serum transferrin isoelectric focusing

In order to verify that the disease was SRD5A3-CDG, we performed serum TIEF (in the older brother) and found an abnormal pattern compatible with SRD5A3-CDG: 5.371% disialotransferrin, 3.546% trisialotransferrin, 86.012% tetrasialotransferrin, and 5.071% pentasialotransferrin. The presence of disialotransferrin and a decreased ratio of trisialotransferrin to diasialotransferrin of 0.66 (normal ratio >1.25) was supportive of a type I pattern.

Considering all evidence together, the patients were diagnosed with SRD5A3-CDG.

## Discussion

The 2 brothers were initially evaluated as having a novel neurologic disorder. We performed genome-wide linkage analysis and found 4 candidate loci. We then launched exome sequencing and found a homozygous nonsense mutation in *SRD5A3* exon 1. The premature translational termination signal was deduced to result in a null protein by deleting 300 residues from the native 318-amino acid protein. The gene has recently been shown to be responsible for SRD5A3-CDG, and all mutations reported so far are truncating [3,4,7,9]. Each of the mutations was found in a single family, except for p.W19X, the mutation in our patients. This mutation was previously reported in the homozygous state in 3 unrelated children [4,6]. The variability and severity of the clinical phenotypes of those patients plus the two patients we present herein with the same homozygous mutation further establish that there is no genotype–phenotype correlation in SRD5A3-CDG and clinical manifestations may vary even within the same family. Both of the previously reported Turkish patients with p.W19X were initially diagnosed with CDG-x [12,13] but later shown to have *SRD5A3* mutation [4]. One of those patients presented with stereotypical dystonic hand movements, truncal hypotonia, cerebellar vermis atrophy, psychomotor retardation, and ophthalmologic abnormalities such as optic atrophy, nystagmus and strabismus [13]. The other patient had floppiness, roving eye movements, and normal social contact in early infancy. Muscle hypotonia developed at the age of 5 months, and severe mental delay, visual impairment, optic atrophy, nystagmus, cerebellar ataxia, and recurrent episodes of reduced activity followed [12]. The third patient reported with p.W19X was a 6-year old Pakistani boy with clinical features of hypertonia, psychomotor retardation, and nystagmus but no ocular findings at the age 5 months [6]. In contrast, our patients had progressive ataxic gait and visual failure since early childhood, and the older brother had a more severe phenotype.

Our findings widen the clinical spectrum of *SRD5A3* mutations, especially at the adult stage. Our patients are around 40 years of age, whereas all previously reported cases of SRD5A3-CDG were children. The most striking clinical features in our patients are ophthalmologic malformations including bilateral optic atrophy, cataracts and visual failure, ataxia and mental status best described as borderline in the younger brother and moderate intellectual disability in the older brother. The phenotype of the younger patient is quite mild and very slowly progressive. The clinical features of our patients and those of the reported patients with SRD5A3 mutation are compared in Table 1. The leading clinical pathology in SRD5A3-CDG seems to include ophthalmic malformations involving both the anterior and posterior segments of the eye, such as ocular coloboma, hypoplasia of the optic nerve, cataract, glaucoma, and variable visual impairment with nystagmus. Both of our patients have severe visual impairment, optic atrophy, and cataracts. Cataracts were reported in only 2 patients with SRD5A3-CDG but in all 3 patients with Kahrizi syndrome [3,4,9]. It was suggested that cataracts could develop later in patients with SRD5A3-CDG, if not already present at infancy [4]. Congenital colobomas, although extremely rare in CDG syndromes, are suggestive of SRD5A3-CDG whenever present, and glaucoma is described in SRD5A3-CDG but not other types of CDG [4,5,14]. Neither of these 2 pathologies was observed in our patients. However, bone spicule pigmentation, which is a fundus feature in retinitis pigmentosa, another rare ocular pathology, was present in both of our patients. Although retinitis pigmentosa has been reported in SRD5A3-CDG [5,14], our patients had a specific form resulting from the migration of pigment-containing cells to perivascular sites in the inner retina. Retinitis pigmentosa might be a late sign in SRD5A3-CDG, and so is optic disc hypoplasia. Only 3 of the 12 patients in the Morava cohort [4] had this feature, whereas both our patients had it. It remains to be seen whether bone spicule pigmentation, observed so far only in our patients, is a frequent late manifestation of SRD5A3-CDG. It was not reported in the 3 siblings with Kahrizi syndrome either; they had cataracts and iris coloboma but not optic atrophy, optic disc hypoplasia, or retinitis pigmentosa.

**Table 1:** **Characteristics of patients with *SRD5A3 *** **mutation**

**Clinical findings**		**Morava cohort**[4]	**Kahrizi cohort**[8]	**Kasapkara patient**[7]	**Our patients [this paper]**
**Origin**					
Turkish		5/12	0/3	1/1	2/2
Iranian		0/12	3/3	0/1	0/2
Baluchi		4/12	0/3	0/1	0/2
Polish		3/12	0/3	0/1	0/2
**Age at diagnosis**		**6 month-12 years**	**Adulthood**	**3.5 years**	**Adulthood**
**Eye findings**					
Ocular coloboma		5/12	2/3	0/1	0/2
Hypoplasia of optic discs		3/12	0/3	1/1	0/2
Optic atrophy		8/12	0/3	1/1	2/2
Nystagmus		12/12	0/3	1/1	2/2
Glaucoma		1/12	0/3	0/1	0/2
Cataract		2/12	3/3	0/1	0/2
Bone spicule pigmentation		0/12	0/3	0/1	2/2
Visual impairment		11/12	?/3	1/1	2/2
Strabismus		NA	0/3	1/1	0/2
Microphthalmia		2/12	0/3	0/1	0/2
**Neurologic findings**					
Muscle hypotonia		10/12	0/3	1/1	0/2
Motor retardation		8/12	3/3	1/1	2/2
Intellectual disability		12/12	3/3	1/1	2/2
Cerebellar vermis atrophy		5/11	0/3	1/1	NA
Global cerebellar atrophy		2/11	0/3	1/1	NA
Cerebellar ataxia		10/11	0/3	1/1	2/2
Spasticity		5/12	0/3	0/1	2/2
Movement disorder		3/12	0/3	0/1	0/2
Stereotypic movements		3/12	0/3	0/1	0/2
Seizures		NA	0/3	0/1	0/2
Microcephaly		NA	0/3	1/1	0/2
Dysmorphism		?/12	3/3	1/1	0/2
**Ichthyosiform skin lesions**		1/12	0/3	1/1	0/2
**Hepato-intestinal disease**		0/12	0/3	0/1	0/2
**Skeletal findings**					
Kyphosis		0/12	3/3	0/1	0/2
Short upper extremities		0/12	0/3	0/1	1/2
Contractures of large joints		0/12	3/3	0/1	0/2
**Congenital cardiac abnormalities**		3/12	0/3	1/1	NA
**Coagulation abnormalities**		7/8	NA	0/1	0/1
**Elevated liver enzymes**		9/10	NA	0/1	0/1
**Microcytic anemia**		8/11	NA	0/1	0/1
**Type I TIEF pattern**		12/12	0/3	1/1	1 + NA/2
**W19X mutation**		2/12	0/2	0/1	2/2

? = the number of patients having the phenotype was not specified; NA = phenotype not assessed.

## Conclusions

We suggest screening for *SRD5A3* mutations in new patients with at least a few of the clinical symptoms of SRD5A3-CDG such as ophthalmologic malformations, visual impairment, cerebellar abnormalities, ichthyosiform skin lesions, abnormal coagulation, and psychomotor retardation, bearing in mind that a normal TIEF pattern does not exclude the diagnosis. The disorder is a clinically recognizable cerebello-ophthalmo-cutaneous syndrome; therefore, even in the absence of abnormal TIEF results, the above-mentioned distinctive features warrant genetic testing for *SRD5A3*[15]. Only one family has been reported with Kahrizi syndrome, an allelic disease; the 3 affected siblings were 40 years or older and had normal TIEF patterns. It was suggested that the TIEF pattern might become normal in adults and the normal pattern in those patients could be due to their older age [16]. The abnormal TIEF pattern in our patient does not support this hypothesis.

Our findings widen the spectrum of the clinical manifestations of SRD5A3-CDG. We emphasize that ocular signs seem to increase with age and patient follow-up with this knowledge in mind could benefit patients diagnosed at a young age.

## Consent

Informed consent was obtained for all 4 participants. The father signed the consent forms for the sons. Written informed consent had been obtained also from the fifty-six unrelated individuals who were screened for the mutation; the samples are part of the anonymized group used for mutation screening in our laboratory. The Boğaziçi University Institutional Review Board for Research with Human Participants approved the study protocol. A copy of the written consent is available for review by the Series Editor of this journal.

## Abbreviations

CDG: Congenital disorder of glycosylation; TIEF: Transferrin isoelectric focusing.

## Competing interests

The authors declare that they have no competing interests.

## Authors’ contributions

BK performed clinical analyses and co-wrote the manuscript; ÖA generated and analyzed genetic data and co-wrote the manuscript; GG performed clinical analyses and co-wrote the manuscript; NB performed ophthalmologic studies and co-wrote the manuscript; AT supervised genetic analyses, obtained funding, coordinated the work and co-wrote the manuscript. All authors read and approved the final version of the manuscript.

## Pre-publication history

The pre-publication history for this paper can be accessed here:

http://www.biomedcentral.com/1471-2350/15/10/prepub

## Supplementary Material

Additional file 1 Multipoint linkage results in chromosomes yielding >2 LOD scores. Click here for file

Additional file 2 **Chromatograms showing homozygous mutation *SRD5A3 *** **c.57G > A (p.W19X) and the reference sequence.** The region was amplified using primers with sequences 5′-GGAGGCCGAGCACTCG and 5′-GCAGCCCGGGAGCAG. Click here for file
