# Supplementary figures and images for: Adult phenotype and further phenotypic variability in SRD5A3-CDG
Source: BMC Med Genet. 2014 Jan 16;15:10. doi: 10.1186/1471-2350-15-10 (PMC3898029; doi:10.1186/1471-2350-15-10)

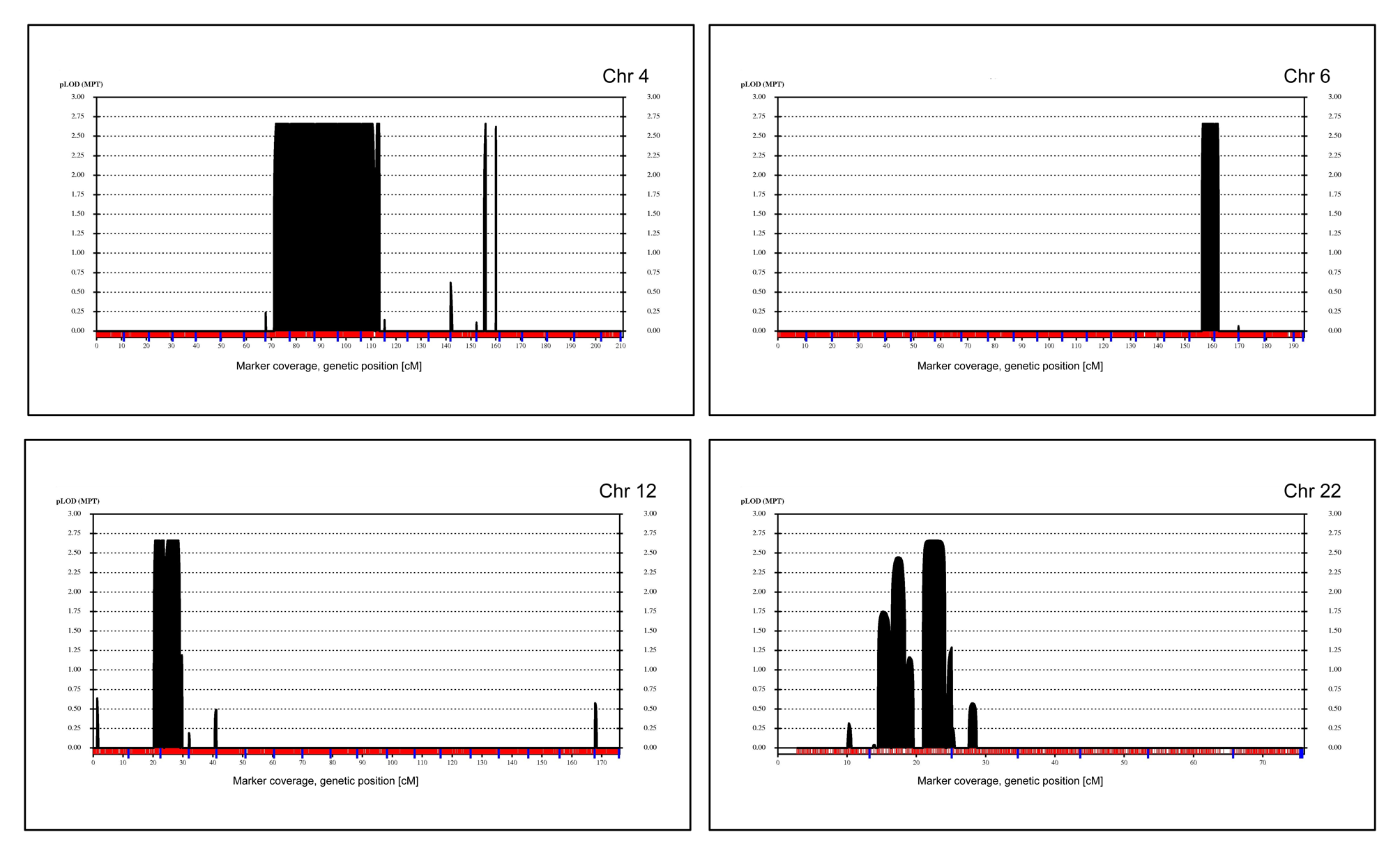

Supplement: Additional file 1 — Multipoint linkage results in chromosomes yielding >2 LOD scores. [file 1471-2350-15-10-S1.tiff]

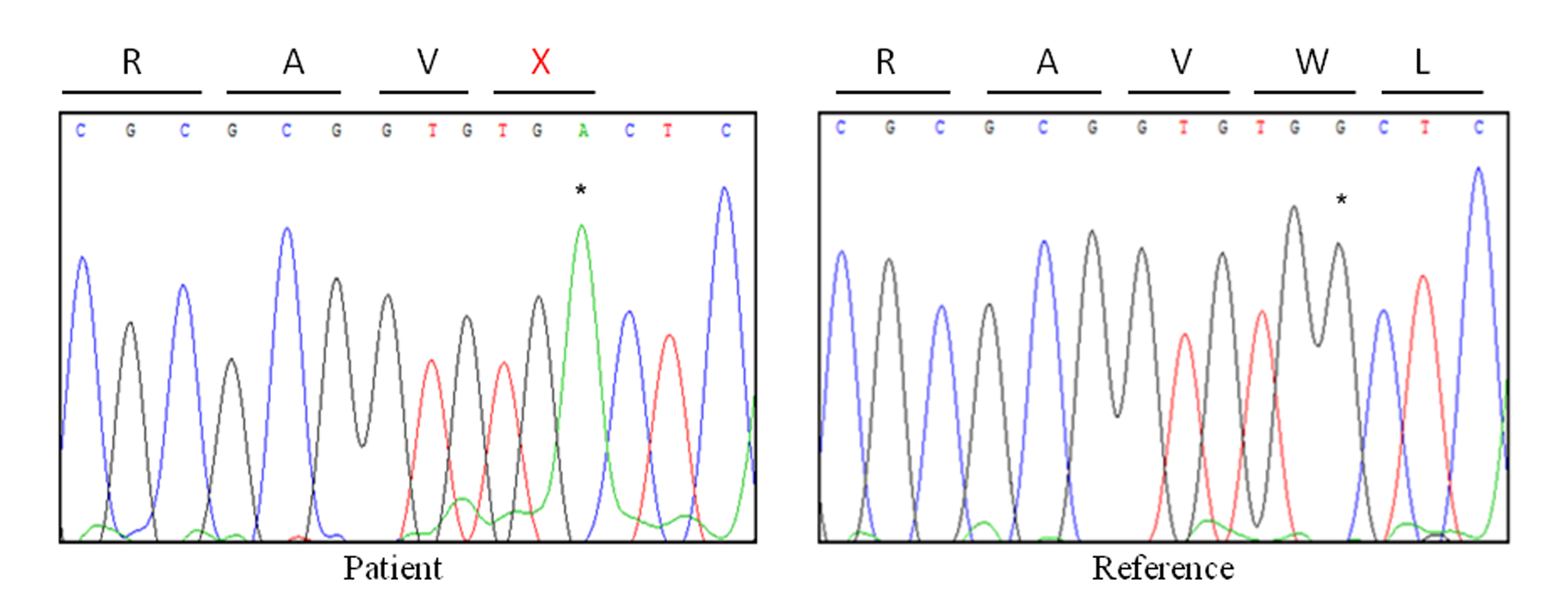

Supplement: Additional file 2 — Chromatograms showing homozygous mutation SRD5A3 c.57G > A (p.W19X) and the reference sequence. The region was amplified using primers with sequences 5′-GGAGGCCGAGCACTCG and 5′-GCAGCCCGGGAGCAG. [file 1471-2350-15-10-S2.tiff]
